# Fluctuations in pedestrian dynamics routing choices

**DOI:** 10.1093/pnasnexus/pgac169

**Published:** 2022-08-27

**Authors:** Alessandro Gabbana, Federico Toschi, Philip Ross, Antal Haans, Alessandro Corbetta

**Affiliations:** Department of Applied Physics, Eindhoven University of Technology, 5600 MB Eindhoven, The Netherlands; Department of Applied Physics, Eindhoven University of Technology, 5600 MB Eindhoven, The Netherlands; CNR-IAC, Via dei Taurini 19, 00185 Roma, Italy; Studio Philip Ross, 5641 JA Eindhoven, The Netherlands; Human Technology Interaction, Eindhoven University of Technology, 5600 MB Eindhoven, The Netherlands; Department of Applied Physics, Eindhoven University of Technology, 5600 MB Eindhoven, The Netherlands

**Keywords:** high-statistics pedestrian dynamics, pedestrians routing, fluctuations, stochastic variational principle, collective behavior

## Abstract

Routing choices of walking pedestrians in geometrically complex environments are regulated by the interplay of a multitude of factors such as local crowding, (estimated) time to destination, and (perceived) comfort. As individual choices combine, macroscopic traffic flow patterns emerge. Understanding the physical mechanisms yielding macroscopic traffic distributions in environments with complex geometries is an outstanding scientific challenge, with implications in the design and management of crowded pedestrian facilities. In this work, we analyze, by means of extensive real-life pedestrian tracking data, unidirectional flow dynamics in an asymmetric setting, as a prototype for many common complex geometries. Our environment is composed of a main walkway and a slightly longer detour. Our measurements have been collected during a dedicated high-accuracy pedestrian tracking campaign held in Eindhoven (The Netherlands). We show that the dynamics can be quantitatively modeled by introducing a collective discomfort function, and that fluctuations on the behavior of single individuals are crucial to correctly recover the global statistical behavior. Notably, the observed traffic split substantially departs from an optimal, transport-wise, partition, as the global pedestrian throughput is not maximized.

Significance Statement Busier urban areas, together with ever increasing expectations on safety and commuting efficiency, call for a deep understanding of human crowd dynamics. Additionally, unusual occurrences, such as high crowd densities during large events or the need to enforce low densities during global pandemics, raise challenging and unexplored demands on the management of human crowds. On the basis of a real-life experiment, we develop a quantitative understanding on the route-choice process for single pedestrians in crowds. We show that single individuals, while optimizing their joint utility, do not optimize the common good, here defined as maximizing the global throughput. Additionally, we demonstrate that fluctuations induced by individual variability are key for qualitatively and quantitatively explaining the observed crowd behavior.

## Introduction

Countless daily-life scenarios entail pedestrians walking towards a common destination and choosing among alternative neighboring routes. Consciously or unconsciously, and in connection with factors such as crowd density, estimated time to destination, path directness (1), perceived comfort/safety, background knowledge, habits, or even aesthetics, each individual selects and walks a preferred route (2–9).

At the individual microscale level, the routing choice has been quantitatively modeled in terms of discomfort functional, $\mathcal {L}$, that individuals seek to minimize (4, 10). From a microscopic description, it is possible to derive the macroscale behavior of a crowd, as in the model introduced by Hughes (11), where the connection between the Fermat principle (i.e. minimization of optical paths) and a macroscopic Eikonal description is used, however, neglecting individual variability.

In this paper, we show that random fluctuations at the single individual scale are key to recover the observed macroscale statistics. We model the decision process via a global (i.e. coupling all pedestrians) variational minimization, showing how crowd flows stem from the combination of the routing decisions operated concurrently by single individuals, comparing with data from a real-life pedestrian tracking campaign.

We consider a crowd of *N* pedestrians, and define a discomfort $\mathcal {L}$ depending on the (perceived) density *ρ*, time to destination *τ*, and path length *λ* (and possibly other quantities), for each single individual. In other words, $\mathcal {L}$ represents a functional defined on the crowd as a whole, entailing the state of each pedestrian.

Understanding qualitatively and quantitatively the physical processes that link (the statistics of) microscopic dynamics and the macroscopic crowding patterns that these generate is an outstanding challenge. On one side, this shares deep connections with active matter physics (12), where optics-like variational principles succeeded at describing dynamics of living agents [e.g. ant trails (13)]. On the other side, physics-based modeling of crowd dynamics retains great relevance in the endeavor to increase safety and comfort of urban infrastructures and large-scale events (14,15).

Among the factors undermining our understanding of crowd flows is the inherent technical challenge of collecting accurate measurements at large spatial and time scales. Thus, the majority of the studies in pedestrian dynamics have leveraged on qualitative simulations (16) via microscopic (17–20) or macroscopic numerical models (21–23). Routing has also been addressed via questionnaires [e.g. (24–26)] or in laboratory conditions (27–31), where it is in general complicated to avoid interfering with the phenomenon at study [see also (32) for a more in-depth review]. Because of this, the role of fluctuations around the average behaviors observed in crowd flows are rarely studied (33,34).

**Fig. 1.**
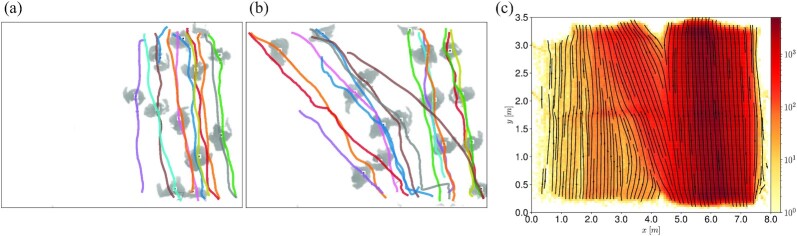
Overview of the experimental data. In (a) and (b) we show two examples of overhead frames recorded by the depth cameras. The depth field in (a) depicts a set of *N* = *N*_A_ = 13 pedestrians all taking the straight path (path A), while in (b) we provide an example of a more balanced pedestrian distribution. The gray shades represent the distance between each pixel and the camera plane (i.e. the elevation from the ground). This type of data allows reliable pedestrian tracking (see “Materials and methods” for details). The automatic tracking output is overlayed as solid colored lines. (c) Heat-map of pedestrians position from the entire dataset. We remark that the colorbar is given in logarithmic scale. The streamlines of the (spatially binned) mean velocity vector are used in order to provide a visual representation of the most probable trajectories.

**Fig. 2.**
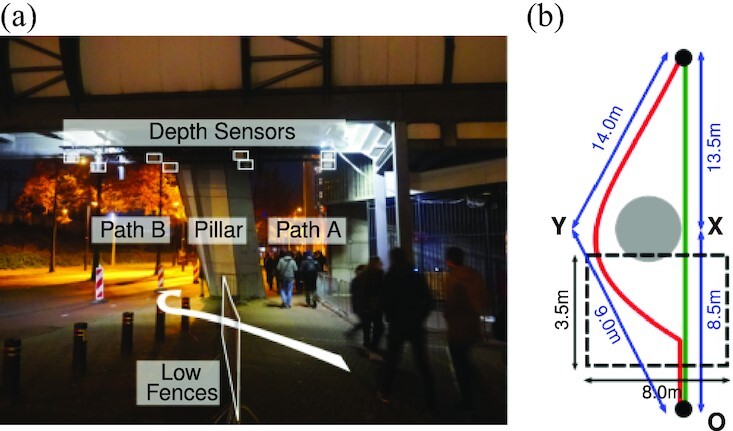
Experimental setup from the viewpoint of a pedestrian walking towards the path bifurcation (a) and sketch of the floor plan (b). A low-fence blockage drives the pedestrian flow towards one same entrance point, while a set of bollards separates the bicycle lane from the adjacent road preventing pedestrians from entering the system from other locations or to exit by an area not covered by cameras. A grid of 4×2 Orbbec depth cameras, hanging below the overpass connecting the Philips Stadium to a nearby train station, is used to collect trajectories within the area marked by dotted black lines in (b).

In this work, we analyze uni-directional pedestrian dynamics around a non-symmetric route bifurcation (Fig. 2), as a paradigm scenario for non-trivial macroscopic routing. We base our analysis on high-accuracy high-statistics individual trajectory data collected during a week-long festival in Eindhoven (The Netherlands), via overhead depth sensing (see Fig. 1 for an example), a methodology, which has emerged in the last decade (35–38) as an effective option to gather accurate tracking data in real-life, even at high pedestrian density (39), while fully respecting individual privacy. This approach enables arbitrarily long tracking campaigns during normal operations of public facilities, and has allowed the analysis of fluctuations and rare events in pedestrian dynamics [e.g. (40–42)].

We study the dynamics around the obstacle in Fig. 2 for different density levels by analyzing the trajectories of about 100,000 individuals. We focus on the statistics of collective routing decisions in dependence on the local crowd density, *ρ*, here considered via the instantaneous number, *N*, of pedestrians in the facility. In what follows we use these two quantities interchangeably, as they can be put in relationship via *ρ* = *N*/*A*_ref_, where $A_{\rm ref} \approx 15.0~\rm {m^2}$ is the reference area effectively used by the pedestrians (see supplementary material).

Under these settings, we show that experimental observations are compatible with realizations of a random process in which the crowd arranges in such a way that the average (estimated) transversal time performs optimally with respect to all other traffic arrangements. In spite of the simplicity of the experimental setup, the observed traffic departs from a global optimal, transport-wise, partition, as the pedestrian throughput is not maximized.

## Measurement campaign

We collected the trajectories used in the analysis presented in this paper during the GLOW light festival, in Eindhoven (The Netherlands), between 2019 November 9 and 16. The festival comprises a city-wide circular route, with mostly uni-directional traffic. We established our measurement setup along the outer perimeter of the Philips Stadium, few hundred meters upstream and downstream from the festival’s light exhibitions. Pedestrians approaching the setup faced the non-symmetric binary choice of bypassing, on either side, a large support pillar (sustaining the stadium grandstands, Fig. 2a). On the right-hand side, the path, from now on referred to as path A, was approximately straight, with free sight of the horizon. The longer path on the left-hand side, path B, partially overlapping a bike lane (partially reserved to pedestrians), was rather curved around and following the pillar base (cf. Fig. 2a and b). The crowd traffic in the area was stemmed by two types of barriers: several bollards placed on the side of path B separated the bicycle lane from the adjacent road, while a low fence directed the flow towards the path bifurcation from a single arrival basin.

The geometrical definition of the length of the two paths, respectively, *L*_B_ and *L*_A_, is subject to a certain degree of arbitrariness, depending on where the initial and final destination points are taken, and on the considered connected trajectories. We shall characterize the geometry of our setup via the non-dimensional constant

(1) $$\begin{eqnarray*}
\lambda _g = \frac{L_{\rm B}}{L_{\rm A}} \gt 1,
\end{eqnarray*}
$$

i.e. the ratio between the two paths lengths.

In order to provide an estimate for λ*_g_*, we consider two different approaches. In the first one, we consider the right-triangle OXY in Fig. 2(b), with vertexes defined by the path midpoint at the entrance of the setup, right at the end of the low fences blockage (“O”), and the midpoints of paths A (“X”) and B (“Y”) across the pillar. In this case it holds λ*_g_* ≈ 1.06. If we restrict ourselves to the area covered by the depth sensors, we can also define λ_*g*_ as a ratio between the length of a typical trajectory in B and in A (cf. Fig. 1c), which provides an overview of the trajectory data as a heat-map of pedestrian positions). Including the uncertainty in the definition of these typical trajectories, it holds 1.3 ≲ λ_*g*_ ≲ 1.4. We shall come back later to the analysis of λ_*g*_ and on how it is perceived by single individuals.

**Fig. 3.**
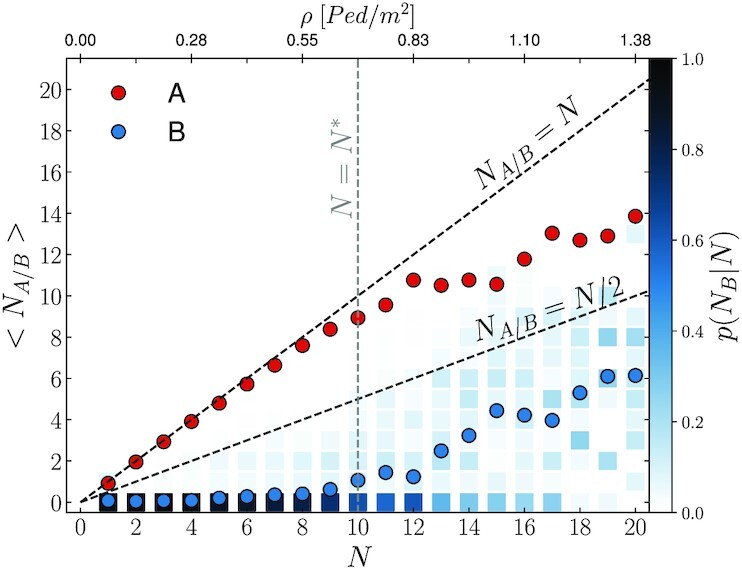
Average number of people taking path A (〈*N*_A_〉, red dots) and B (〈*N*_B_〉, blue dots) as a function of the global pedestrian count *N*. We observe that until *N* < N* = 10, on average less than one person opts to travel along path B. Above the N* threshold, people start making systematic use of path B and both diagrams exhibit a clear change. The blue colorbars provide a visual representation of the probability distribution *P*(*N*_B_|*N*) of number of people taking path B, conditioned to the global pedestrian count *N*. Even when *N* > N*, configurations in which no pedestrian walks on path B are frequent.

In low density conditions, pedestrians opt for path A in the greatest majority of cases (e.g. for *N* <10 path A is preferred in $\approx 95 \%$ of cases). This is shown in Fig. 3, where we report the local average occupancy of the two paths, respectively 〈*N*_A_(*N*)〉 and 〈*N*_B_(*N*)〉, calculated on uncorrelated frames as a function of the instantaneous count *N* (see supplementary material). As the number of pedestrians increases, we observe that path B “activates” as people start to systematically opt for it. We denote with N* the global pedestrians count at which path B activates, which we define as the minimum value of *N* at which, on average, at least one person takes path B; in our setup N* = 10.

The local occupancy of paths A and B exhibits clear slope changes around N*. In flow terms, N* corresponds to the transition from a strongly unbalanced distribution, in which rarely a pedestrian is found walking along path B, towards a more balanced A–B load partition.

Fig. 3 includes a visual representation of the conditioned probability of the occupancy of path B, given the global pedestrian count *N*, i.e. *P*(*N*_B_|*N*). Even when *N* is much larger than N*, *P*(*N*_B_|*N*) is bi-modal: path B remains often empty. For instance, at *N* = 20, we observe that in about $10 \%$ of the cases, pedestrians choose to walk only along path A. This observation points to the presence of a collective dynamics in which pedestrians at times follow others rather than attempting to optimize the flow partitioning. So, how do pedestrian choose the path? A quantitative modeling of this peculiar aspect will be the focus of our analysis in the coming sections.

**Fig. 4.**
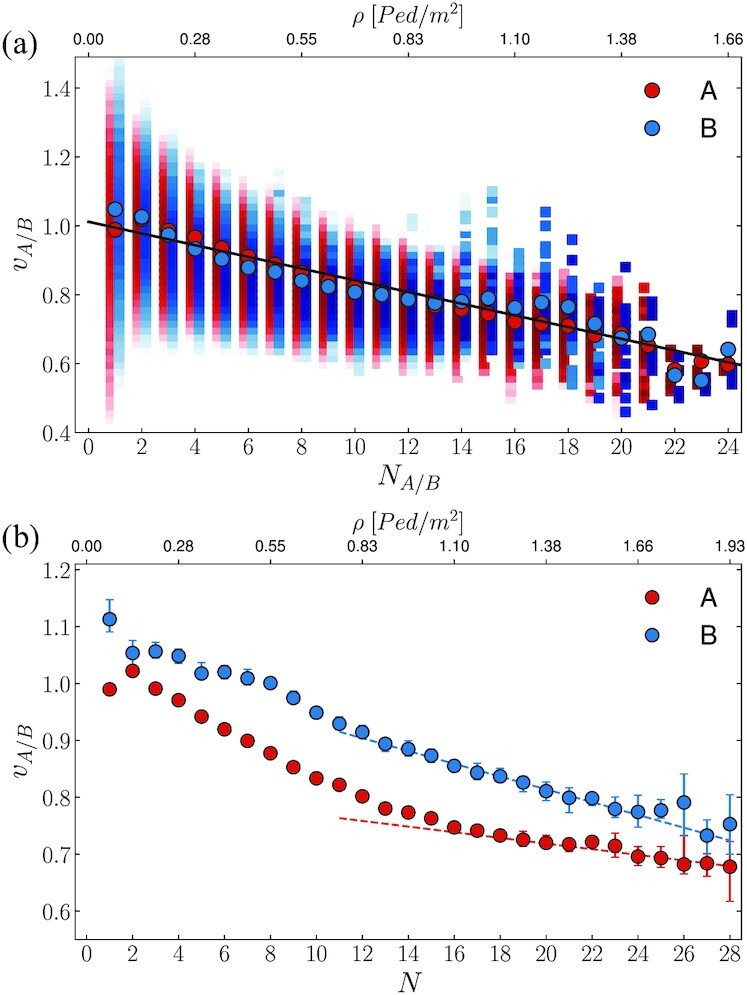
Fundamental velocity diagrams. (a) Local velocity as a function of the number of people present along path A (red) and path B (blue). Dots represent the average values, while colorbars synthesize the probability distribution functions (PDFs). The black solid line provides a linear fit of the local velocity diagrams under the reasonable assumption that the same fundamental diagram applies to both paths. (b) Global velocity as function of the total number of people in the system. We highlight an evident change in the slope of the diagram for both path A and path B for *N* > N*, which we model (dotted lines) with a re-parametrization of the local velocity diagram. (See main text for details).

Different global and local pedestrian count levels (i.e. in either path A or B) reflect on different average walking velocities. Fig. 4(a) reports the (average) local walking velocity along paths A and B as a function of the local pedestrian count: *v_J_* = *v_J_*(*N_J_*), with *J* ∈ {A, B}. In turn, Fig. 4(b) reports how velocity depends on the global pedestrian count. These correspondences between velocity and the density/pedestrians-count, generally dubbed fundamental diagrams, are the most commonly adopted tool for macroscopic descriptions of vehicular and pedestrian traffic [cf. e.g. (43–46)].

As the number of pedestrians increases, the average walking velocity decreases. Consistently with studies conducted in comparatively low-density regimes (44), we observe, on average, a linear decay trend in the local fundamental diagrams:

(2) $$\begin{eqnarray*}
\langle v_{J}(N_{J}) \rangle = v_0 - \kappa ~ N_{J},
\end{eqnarray*}
$$

where *v*_0_ is the “free-stream velocity” in the zero-density limit and *κ* fixes the diagram slope. We assume the local fundamental diagram to be the same, for people walking in paths A and B. We have verified this by performing a fit for the parameters *v*_0_ and *κ*, independently, for the two sets of pedestrians walking either of the two paths and observing no significant differences. In Fig. 4(a), we show with a solid line the best fit on the overall dataset, given by $v_0 \approx 1.012~\rm {m/s}$, $\kappa \approx 0.017~\rm {m/s \cdot 1/ped}$, with the coefficient of determination *R*^2^ ≈ 0.93. Fig. 4(a) additionally reports the full conditioned probabilities *P*(*v_J_*|*N_J_*) that highlights velocity fluctuations, *ϵ*, around the average. We shall address these as independent with respect to the pedestrian count *N*, and additive with respect to the average velocity, in particular

(3) $$\begin{eqnarray*}
\epsilon \sim \mathcal {N}(\mu =0, \sigma =0.15),
\end{eqnarray*}
$$

where $\mathcal {N}$ is the Gaussian distribution and the variance *σ* has been estimated from the experimental data (see Supplementary Material). The global fundamental diagrams, 〈*v_J_*〉 = 〈*v_J_*(*N*)〉 in Fig. 4(b), contrarily to their local counterparts, display qualitative and quantitative differences between the routes. For any value of *N*, the average walking velocity in path B is higher than in A:

(4) $$\begin{eqnarray*}
\langle v_{\rm B}(\mathit{ N}) \rangle \gt \langle v_{\rm A}(\mathit{ N}) \rangle ,\quad \forall \mathit{ N}.
\end{eqnarray*}
$$

Second, we observe a change in slope, ∂_*N*_〈*v_J_*(*N*)〉, around *N* ≈ N* (we employ the symbol ∂_*N*_ for the partial derivative ∂/(∂*_N_*)). For *N* < N*, the global diagram for path A coincides with its correspondent local diagram:

(5) $$\begin{eqnarray*}
\langle v(\mathit{ N}_{\rm A}) \rangle \approx \langle v_{\rm A}(\mathit{ N}_{\rm A}) \rangle ,\quad \mathit{ N} \lt \mathit{ N}^{*}.
\end{eqnarray*}
$$

This is natural since, in this range, *N*_A_(*N*) ≈ *N* holds (Fig. 3). On path B, the velocity as a function of *N* decreases linearly, yet at a smaller rate than −*κ* (i.e. ∂_*N*_〈*v*_B_(*N*)〉 < −*κ*). When *N* is small, path B is rarely employed (cf. PDF of the local density *N*_B_ in Fig. 3). This allows pedestrians to easily walk at their preferred walking speed (i.e. the free stream velocity *v*_0_).

Conversely, when *N* > N*, the activation of path B yields *N*_A_(*N*) < *N*. This reflects in the slower decay of 〈*v*_A_(*N*)〉 as *N* increases in comparison with the local counterpart:

(6) $$\begin{eqnarray*}
\partial _\mathit{ N} \langle v_{\rm A}\mathit{ (N)} \rangle \lt -\kappa ,\quad \mathit{ N} \gt \mathit{ N}^{*}.
\end{eqnarray*}
$$

We can reconstruct the global fundamental diagram from the local diagram by considering *N_J_* = *N_J_*(*N*). This yields

(7) $$\begin{eqnarray*}
\partial _\mathit{ N} \langle v_{J}\mathit{ (N)} \rangle \approx - \kappa \partial _\mathit{ N} \mathit{ N}_{J}\big |_\mathit{ N},\quad \mathit{ N} \gt \mathit{ N}^{*},
\end{eqnarray*}
$$

which satisfies Eq. (6) since ∂_*N*_*N_J_* <1 holds in the considered regime (cf. Fig. 3; see the dotted lines included in Fig. 4b).

We conclude this section turning our analysis to the pedestrians flow, which we define as

(8) $$\begin{eqnarray*}
\phi (\mathit{ N}) = \langle v_{\rm A}(\mathit{ N}_{\rm A}) \rangle \mathit{ N}_{\rm A}(\mathit{ N}) + \langle v_{\rm B}(\mathit{ N}_{\rm B}) \rangle \mathit{ N}_{\rm B}(N) .
\end{eqnarray*}
$$

By making use of the fundamental velocity diagram, we can conveniently define a theoretical upper bound and lower bound for Eq. (8), which are found respectively in correspondence of the optimal partitioning *N*_A_(*N*) = *N*/2, and the most unbalanced case *N*_A_(*N*) = *N* (or likewise *N*_A_(*N*) = 0). The above holds under the assumption that the section of path A equals that of path B, which is approximately true in our setup. Combining this information with the velocity fundamental diagram in Eq. (2), we can define

(9) $$\begin{eqnarray*}
\phi _{\rm ideal}(\mathit{ N}) = v_{\rm A} \left(\frac{\mathit{ N}}{2}\right) \mathit{ N} , \quad \phi _{\rm unbalanced}(\mathit{ N}) = v_{\rm A}(\mathit{ N}) \mathit{ N} .
\end{eqnarray*}
$$

**Fig. 5.**
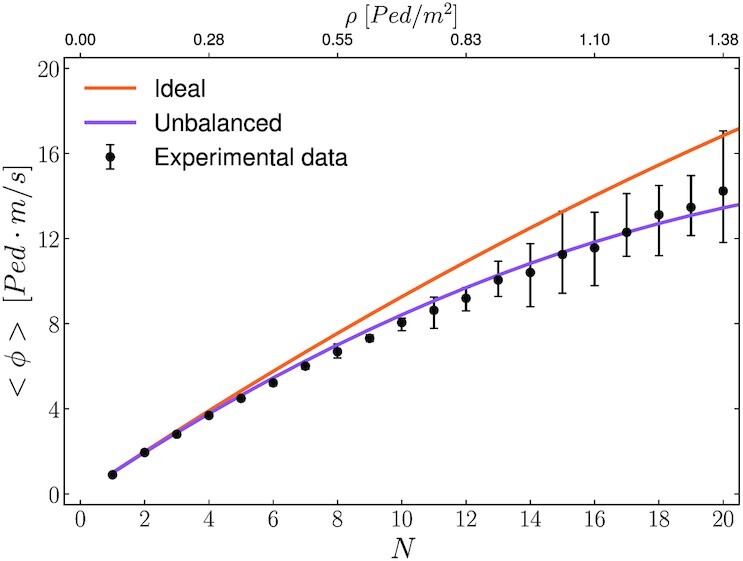
Average pedestrian flow (Eq. 8) as a function of the pedestrian count. The solid lines represent the theoretical maximum (orange color) and minimum (purple color) case scenarios. We observe that experimental results (black dots) on average closely follow the flow of the most unbalanced case. The error bars have been obtained by dividing the data into 10 bins, with the extrema of the error bars representing the minimum and maximum average value per bin.

In Fig. 5, we compare the experimental data with the modeling from Eq. (9). The slope *κ* determines the differences between the upper bound and lower bound, which in the density range considered are at most $20 \%$. Nevertheless a clear trend emerges, with the experimental data closely following (on average) the flow of the highly unbalanced configuration; this provides clear-cut evidence for pedestrians not managing to maximize the global throughput, despite the simplicity of the setup.

On these bases, in the following section, we introduce a model for studying the routing behavior and the features arising at the transition around *N* ≈ N*, and where we assume that pedestrians aim at optimizing their benefit (perceived travel time to destination).

## Results

### Model

We aim at a minimal model exposing the underlying mechanisms involved in the routing decision.

Although a time-dependent model for the probability of choosing either paths, already pursued by the same authors (47), appears like a natural choice, its success is enslaved to the comprehension of the complex time correlation characterizing the choice process, or to phenomenological data-fitting (47,48).

The short duration of the festival, the relatively limited number of tracking hours, and the high variability in the crowd make a time correlation analysis extremely challenging. Therefore, aiming at a bottom-up physical model, we pursue a time-independent approach.

We consider a simulated crowd of *N* pedestrians indexed by *i* = 1, …, *N* about to cross the experiment area in Fig. 2. We allow each individual to choose between path A or B in awareness of the choice of others. This gives configurations *c* in the form of

(10) $$\begin{eqnarray*}
c = (J^{(1)}, J^{(2)}, \ldots ,J^{(N)}) ,
\end{eqnarray*}
$$

where *J*^(*i*)^ equals A or B depending on the path selected by the the *i*th pedestrian.

Let $v^{(i)}_{J}=v^{(i)}_{J}(N_{J})$ be the walking velocity of the *i*th pedestrian on path *J* as a function of the local density *N_J_*, i.e. the local fundamental diagram [cf. (2), (3)]. We define the *perceived* travel time

(11) $$\begin{eqnarray*}
\tau ^{(i)}_{J} = g^{(i)}_{J}\left(\frac{L_{J}}{v^{(i)}_{J}(N_{J})}\right)
\end{eqnarray*}
$$

in either paths to be a key variable in the A vs. B choice; here $g^{(i)}_{J}(\cdot )$ is a function mapping the actual “geometric” travel time $L_{J}/v^{(i)}_{J}$ to the perceived one. The expression of $g^{(i)}_{J}$ will be discussed later on.

We consider a variational framework in which path choices are such that the minimum for the crowd-level functional

(12) $$\begin{eqnarray*}
\mathcal {L}= \mathcal {L}(\tau ^{(1)}_{J_1},\ldots ,\tau ^{(N)}_{J_N}),
\end{eqnarray*}
$$

is attained. We consider a dynamics in which pedestrians arrange to reduce the total *perceived* travel time:

(13) $$\begin{eqnarray*}
\mathcal {L}= \sum _{i=1}^{N}\tau ^{(i)}_{J_i}.
\end{eqnarray*}
$$

Defining the discomfort functional $\mathcal {L}$ is the modeling endeavor: the choice is not unique, yet Eq. (13) gave us the best agreement with observations; the interested reader will find a comparison with a model adopting a different choice for $\mathcal {L}$ in the supplementary information.

To summarize, we consider a system that takes the configuration c* ∈ Γ for which

(14) $$\begin{eqnarray*}
\min _{c\in \Gamma }\left[ \sum _{i=1}^{N}\tau ^{(i)}_{J_i} \right] = \min _{c\in \Gamma }\left[ \sum _{i=1}^{N} g^{(i)}_{J}\left(\frac{L_{J}}{v^{(i)}_{J}(N_{J})}\right)\right],
\end{eqnarray*}
$$

with Γ representing the full set of 2^*N*^ distinct configurations, and with the individual velocities [cf. Eq. 2] satisfying

(15) $$\begin{eqnarray*}
v^{(i)}(N_{J}) = v_0 - \kappa ~ N_{J} + \epsilon ^{(i)},
\end{eqnarray*}
$$

with *ϵ*^(*i*)^ independent and identically distributed realization of Eq. (3).

Notably, the case *ϵ*^(*i*)^ = 0, $g^{(i)}_{J}(x)=x$ (i.e. deterministic velocity, and no fluctuations in the perception of the path-length) reduces to a Hughes-like model (1), and has the analytic solution in terms of optical lenghts:

(16) $$\begin{eqnarray*}
\frac{L_{\rm A}}{v_{\rm A}(N_{\rm A})} = \frac{1}{\sqrt{\lambda _g}} \frac{L_{\rm B}}{v_{\rm B}(N_{\rm B})},
\end{eqnarray*}
$$

where we have dropped the index *i* since pedestrians are now indistinguishable from each other. The above implies the following expression for *N*_A_ = *N*_A_(*N*):

(17) $$\begin{eqnarray*}
N_{\rm A}(N) = \min \left\lbrace N , \frac{\kappa N + v_0(\sqrt{\lambda _g} -1)}{\kappa ( \sqrt{\lambda _g} + 1 )} \right\rbrace.
\end{eqnarray*}
$$

Moreover, from Eq. (16), we can define a link between λ_*g*_ and the local velocity of pedestrians in paths A and B: $\lambda _g = \left( \frac{v_{\rm B}(N_{\rm B})}{v_{\rm A}(N_{\rm A})} \right)^2$. The above expression suggests an alternative pathway for measuring λ_*g*_ directly from experimental data. To this aim, we introduce the instantaneous quantity

(18) $$\begin{eqnarray*}
\lambda _p(t) = \left( \frac{\hat{v}_{\rm B}(t)}{\hat{v}_{\rm A}(t)} \right)^2,
\end{eqnarray*}
$$

where $\hat{v}_{\rm B}$ (resp. $\hat{v}_{\rm A}(t)$) indicates the average walking speed of pedestrians in path B (resp.A) measured at time *t*.

**Fig. 6.**
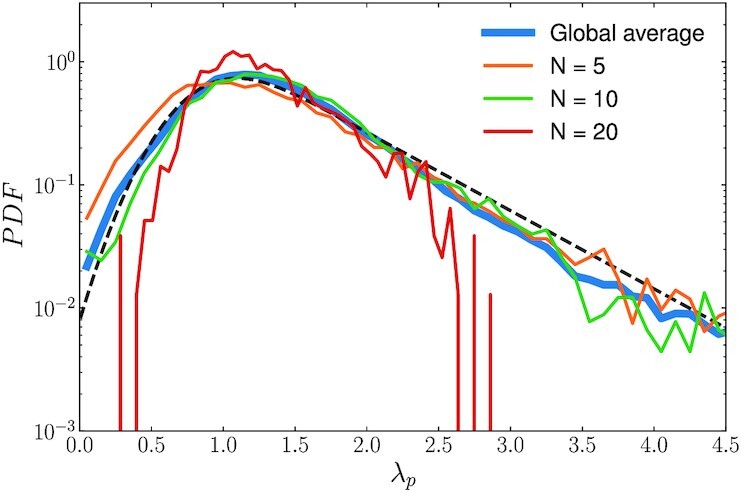
*PDF* of the perceived path length ratio λ_*p*_ [cf. (18)]. We report the distribution for for three different global density values, respectively at low (*N* = 5), intermediate (*N* = 10), and high (*N* = 20) density values. The blue line shows the *PDF* obtained considering the overall dataset, with the black dotted line representing a fit making use of an exponentially modified Gaussian (see Eq. 20), with mean *μ* = 0.77 and standard deviation *σ* = 0.30, and an exponential distribution with scale parameter β = 0.68.

In Fig. 6, we show the *PDF* of *λ_p_*, for the overall dataset, and also conditioned on a few selected values of *N*; we report three representative examples at low, intermediate and large density values (PDFs are restricted to meaningful cases *N*_A_, *N*_B_ > 0). Two aspects emerge. The modal value, mode(λ_*p*_) ≈ 1.2, of the distributions is independent on the global pedestrian count *N*, consistently with the deterministic model in Eq. (16). While, mode(λ_*p*_) is comparable with the estimates of λ_*g*_ provided in the previous section, we observe that the distributions for λ_*p*_ are skewed and carry heavy tails, in particular at low densities.

These are due to observed configurations strongly departing from the deterministic optimum in Eq. (16). Right tails corresponds to cases in which many pedestrians walk along path A even though it might have been less costly (in $\mathcal {L}$ terms) to take B. This can be motivated considering that opting for path B involves traveling around an obstacle, which hides the horizon and to invade the (temporarily closed) bike lane.

The variance of the distributions decreases with the global density. This is consistent with the fact that for *N* > N* the load between A and B gets (on average) increasingly balanced, conversely, the herding becomes weaker (see Fig. 3).

In the next section, we compare Monte Carlo simulations of the dynamics considering various models for λ_*p*_, which we integrate in Eqs. (13) and (14) by defining the conversion functions $g^{(i)}_{J}(\cdot )$ as *i*-independent (i.e. pedestrian-independent) rescaling factors

(19) $$\begin{eqnarray*}
&& g_{A}\left(\frac{L_{\rm A}}{v_{A,i}(N_{\rm A})}\right) = \frac{L_{\rm A}}{v_{A,i}(N_{\rm A})}, \nonumber \\
&& g_{B}\left(\frac{L_{\rm B}}{v_{B,i}(N_{\rm B})}\right) = \frac{\lambda _p}{\lambda _g}\frac{L_{\rm B}}{v_{B,i}(N_{\rm B})} = \lambda _p\frac{L_{\rm A}}{v_{B,i}(N_{\rm B})},
\end{eqnarray*}
$$

Following the *PDF* in Fig. 6, we fit λ_*p*_ with an *N*-independent exponentially modified Gaussian distribution (i.e. the sum of independent normal and exponential random variables):

(20) $$\begin{eqnarray*}
\lambda _p = X + Y,
\end{eqnarray*}
$$

where $X \sim \mathcal {N}(\mu = 0.77, \sigma = 0.30)$ and *Y* ∼ exp(β = 0.68), where β is the scale parameter of the exponential distribution; observe that the expected value is given by *E*[*X* + *Y*] = *μ* + β = 1.45.

### Numerical results

While the deterministic version of the model offers access to a simple analytic solution Eq. (16), this is not the case for the non-deterministic model Eqs. (14), (15), (19), and (20). Therefore, to perform our analysis and compare with measurements we rely on Monte Carlo simulations to identify the statistics of optimal configurations in dependence on the stochastic terms considered: c* = c*(λ_*p*_, *ϵ*^(1)^, …, *ϵ*^(*N*)^).

**Fig. 7.**
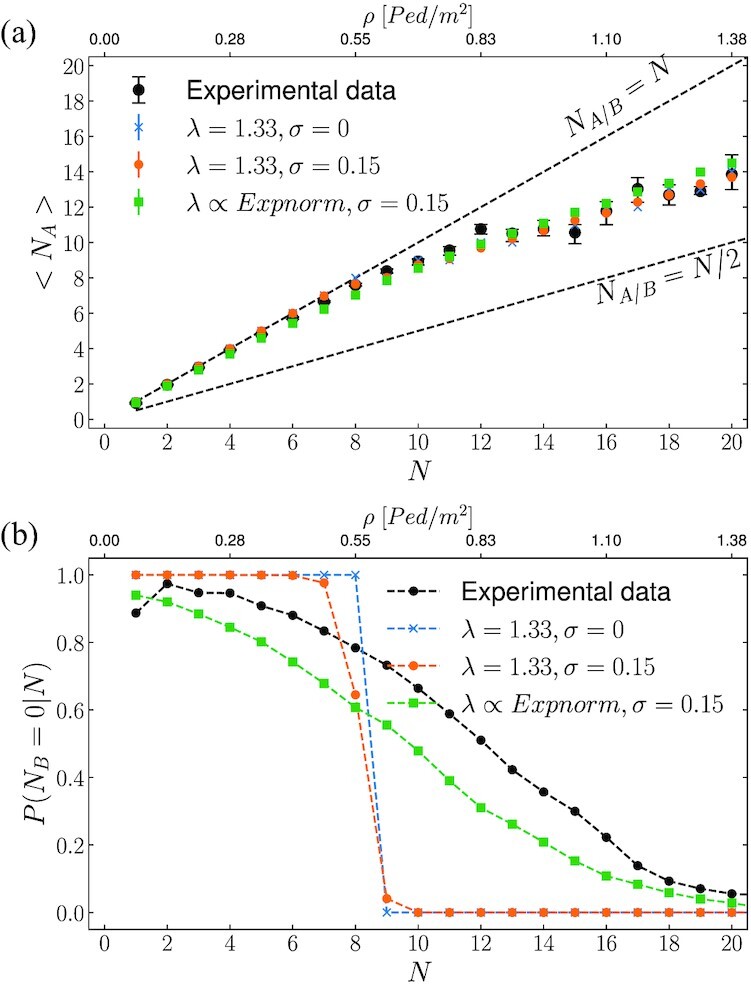
Comparison of numerical results from simulations against experimental data. (a) <*N*_A_(*N*)>: the average number of people taking path A as a function of the global pedestrian count *N*. (b) *P*(*N*_B_ = 0|*N*), the Bernoulli probability of observing configurations in which no pedestrians walk across path B, conditioned on the global pedestrian count *N*. It is evident that fluctuations on the perceived path length allow a more realistic description of the transition around N*, as shown in (b), still correctly capturing the average behavior, as shown in (a).

In Fig. 7(a) we compare the model and experimental data on the average number of people taking path A, <*N*_A_>, conditioned to the global density *N*. The numerical results provide a good description of the measurements, and, in particular, they capture the transition at N*. The model is capable of reproducing, with very good accuracy, also the footprints of the herding effect: this is shown Fig. 7(b), reporting the (Bernoulli) probability of observing exactly zero pedestrians walking along path B, conditioned to *N* (i.e. *P*(*N*_B_ = 0|*N*)). In order to obtain a good agreement between experimental data and simulations we have tuned the parameters of the distribution from which λ_*p*_ is drawn; the results presented in this section make use of Eq. (20) with $X \sim \mathcal {N}(\mu = 1.15, \sigma = 0.20)$ and *Y* ∼ exp(β = 0.33).

With the aim of exposing the role of random fluctuations, in Fig. 7 we show the results obtained by employing a fully deterministic model (i.e. with a deterministic fundamental velocity diagram, *ϵ*^(*i*)^ = 0, and with a constant value for λ_*p*_) as well as a case in which we allow fluctuations in the velocity, but no stochasticity on λ_*p*_.

The deterministic model well captures the average routing choice performed by pedestrians, as shown in Fig. 7(a). On the other hand, it also highlights a sharp transition at N* (see Fig. 7(b)): when *N* < N*, all pedestrians systematically route for path $\rm A$, while for *N* > N*, the optimal configurations do not allow for cases in which exactly zero pedestrians are found walking along path B.

When including fluctuations in the velocity (orange curves), we obtain two relevant effects connected to each other. The walking speed variability creates (rare) optimal configurations c* with pedestrians on path B, even at density values *N* < N*; this effect, only slightly visible in Fig. 7(b), becomes more pronounced as the variance associated to *v*_0_ is increased, in turn leading to a smaller predicted value for N*.

**Fig. 8.**
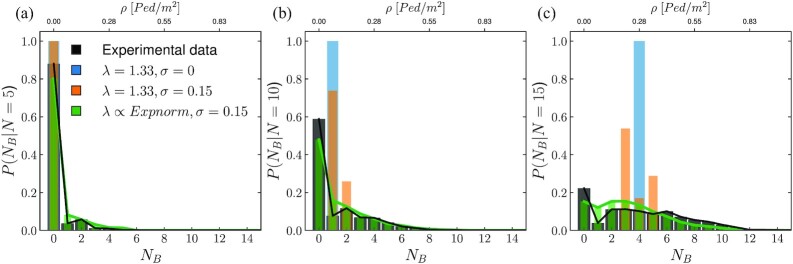
PDF of number of people in path B, *N*_B_, conditioned on the global count *N*. We report three examples, representative of three different levels of density: from left to right *N* = 5, 10, and 15, respectively. The experimental data (black bars) are compared against the results obtained employing three different models: in blue; the results obtained employing a deterministic model in which individual fluctuations are neglected, in orange; the results of a stochastic model accounting for fluctuations in individuals free-stream velocities, and finally, in green; the results of a stochastic model accounting for fluctuations in both free-stream velocities and path length perception for single individuals.

Introducing fluctuations in the model is crucial to provide an accurate description of the variability observed in the experimental data. This is clearly shown in Fig. 8, where we plot the PDF for the number of people walking along path B, conditioned to the global count *N*. The figure reports three representative examples corresponding to different values of *N*. For low density values, the PDFs show a strong peak at *N*_B_ = 0. As *N* increases, the bins corresponding to *N*_B_ > 0 start populating and, eventually, a bi-modal distribution emerges, together with an increased variability in the observed configurations.

Comparing once again the numerical results with the experimental data we can observe that the deterministic model cannot be used to describe the variability observed in the data, although it can provide an approximation to the average of PDFs. While introducing fluctuations in the pedestrians velocity only slightly increases the variability of the PDFs for *N* > N*, it is only with the superposition of the herding effect (green curves) that the model is able to provide a good description of the PDFs. Remarkably, we are able to reproduce to good accuracy also the spikes in correspondence of *N*_B_ = 0 at large values of *N*.

In conclusion, we have shown that fluctuations are crucial for giving a realistic representation of the behaviors observed around *N* ≈ N*.

## Discussion

In this work, we have exposed the crucial role played by individual variability in pedestrians routing choices. Fluctuations emerge as a key element in explaining (intermittent) transitions from highly unbalanced to more balanced configurations which, on average, lead to a sub-optimal traffic partitioning.

We have based our analysis on a large dataset of pedestrian trajectories collected during an unprecedented high-accuracy pedestrian tracking campaign. We have considered a simplified setup in which a unidirectional pedestrian flow is confronted with a binary choice between two paths, presenting marginal differences in terms of length and geometrical complexity. We regard this setup as an excellent prototype for more complex scenarios where, e.g. the trajectory of a pedestrian results from the concatenation of multiple binary choices.

We have developed a time-independent variational model, which has allowed to successfully describe, both at a qualitative and quantitative level, the observed macroscopic patterns. Our modeling shows that we can explain the crowd behavior by considering a crowd-level minimization of the *estimated traveling time*, and accounting for the inherent stochasticity of (i) the walking speed of each single pedestrian, and (ii) the estimation of the path length.

In spite of the simplicity of the experimental setup, our analysis highlights a systematic deviation from global optimum configurations, leading to the global pedestrian throughput not being maximized. Additionally, further and sudden capacity drops appear due to the occurrence of herding behaviors—in which the crowd blindly opt for a highly sub-optimal “follow the lead” choice, rather than completely leveraging the allowed walking space. We remark that in our analysis we use the word “herding” in a broad sense, including both following effects as well as the presence of social groups attending the event. This choice is due to the fact that groups cannot be easily identified in the relatively short-scales of the experiment presented in this work, something on the other hand possible when observing trajectories in a much larger space/time frame (49).

These results clearly point towards the necessity of implementing efficient crowd management measures in order to increase comfort and safety, based on a deeper understanding of the physics of crowds.

To conclude, in this work we have introduced an approach for analyzing the statistics and the efficiency of macroscopic crowd configurations, highlighting an intrinsic sub-optimality in the natural flow of pedestrians, while setting a standard for effective quantitative modeling.

## Supplementary Material

pgac169_Supplemental_File

## Data Availability

The dataset with the pedestrian trajectories used in our analysis is available at https://doi.org/10.5281/zenodo.7007358, whereas examples and processing scripts can be found at https://github.com/crowdflowTUe/2022_fluctuations_in_routing_glow.

## Preprints

A preprint of this article is published at [arXiv:2202.02108].
