# Supplementary material for: Fluctuations in pedestrian dynamics routing choices
Source: PNAS Nexus. 2022 Aug 27;1(4):pgac169. doi: 10.1093/pnasnexus/pgac169 (PMC9802426; doi:10.1093/pnasnexus/pgac169)
Supplement: pgac169_Supplemental_File [file pgac169_supplemental_file.pdf]

PNAS Nexus, 2022, pp. 1–4

doi: 10.1093/pnasnexus/pgac169
Advance Access Publication Date: 27 August 2022
Supplementary Material

SUPPLEMENTARY MATERIAL

Supplementary material for: "Fluctuations in pedestrian dynamics routing choices"

Alessandro Gabbana,[a] Federico Toschi,[a,b] Philip Ross,[c] Antal Haans[d] and Alessandro Corbetta[a,*]

[a]Department of Applied Physics, Eindhoven University of Technology, 5600 MB Eindhoven, The Netherlands, [b]CNR-IAC, Via dei Taurini 19, 00185 Roma, Italy, [c]Studio Philip Ross, 5641 JA Eindhoven, The Netherlands and [d]Human Technology Interaction, Eindhoven University of Technology, 5600 MB Eindhoven, The Netherlands

[*]Corresponding author: a.corbetta@tue.com

Abstract

Routing choices of walking pedestrians in geometrically complex environments are regulated by the interplay of a multitude of factors such as local crowding, (estimated) time to destination, (perceived) comfort. As individual choices combine, macroscopic traffic flow patterns emerge. Understanding the physical mechanisms yielding macroscopic traffic distributions in environments with complex geometries is an outstanding scientific challenge, with implications in the design and management of crowded pedestrian facilities. In this work, we analyze, by means of extensive real-life pedestrian tracking data, unidirectional flow dynamics in an asymmetric setting, as a prototype for many common complex geometries. Our environment is composed of a main walkway and a slightly longer detour. Our measurements have been collected during a dedicated high-accuracy pedestrian tracking campaign held in Eindhoven (The Netherlands). We show that the dynamics can be quantitatively modeled by introducing a collective discomfort function, and that fluctuations on the behavior of single individuals are crucial to correctly recover the global statistical behavior. Notably, the observed traffic split substantially departs from an optimal, transport-wise, partition, as the global pedestrian throughput is not maximized.

Experimental Setup

The trajectories used in the analysis presented in this work have been collected during the 2019 edition of the GLOW light festival in Eindhoven (The Netherlands). The experiment lasted the entire duration of Glow 2019, from November 9th until November 16th, 2019. The tracking was performed during the festival opening hours, every day from 18:00 until 00:00. The data collected on the 14th of November has not been included in the analysis, since on that day the experimental setup was modified in order to evaluate the impact of changing the lighting conditions on the crowd dynamic.

In Fig. S1 we show the pedestrian count as a function of time, with a inset highlighting the fluctuations in the number of pedestrians observed in a 15 minute window.

Pedestrian Sensing

We collected raw depth images of a walkable area of about $30\,m^2$ via 8 Orbbec Persee sensors attached underneath a pedestrian overpass, and arranged in a 4x2 grid (see Fig. 2 in the main text). The depth cameras acquired images at a frame rate of 30 Hz. The trajectories have been obtained from the raw depth images via the Height-Augmented Histogram of Oriented Gradients algorithm (HA-HOG) (see [S4] and [S2, S1] for detailed explanations on pedestrian tracking via depth sensor grids).

In Fig. S2 we show a depth map example with the trajectories resulting from the tracking of the 10 pedestrians in overlay.

The black dotted lines represent the raw trajectories, obtained by applying the HA-HOG algorithm. Solid lines represent the result of applying a Savitzky-Golay filter [S5] to the trajectories. This operation allows to reduce the level of noise and discontinuities which affect the calculation of derivatives, used, for example, to estimate the instantaneous velocity of pedestrians:

$$\boldsymbol{v}(t) = \left(\frac{\tilde{s}_x(t+\Delta t) - \tilde{s}_x(t)}{\Delta t}, \frac{\tilde{s}_y(t+\Delta t) - \tilde{s}_y(t)}{\Delta t} \right) \tag{S1}$$

© The Author 2022. Published by Oxford University Press. All rights reserved. For permissions, please e-mail: journals.permissions@oup.com

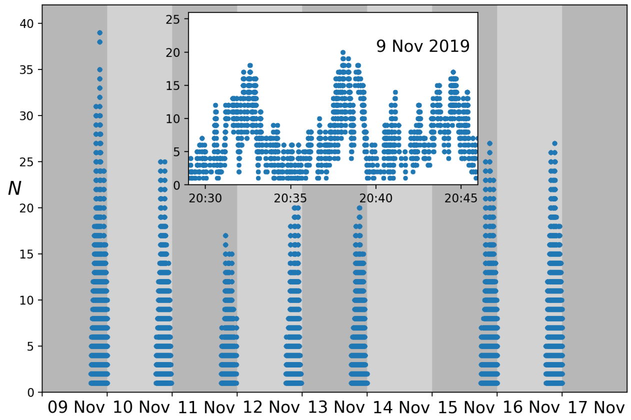

Fig. S1. Pedestrian global count, N, as a function of time during the 8 days of the festival. The inset shows the fluctuations in the number of pedestrians observed in a 15 minute window around 20.40 during the first day of the experiment, highlighting the presence of both low and high density scenarios. The data collected on the 14th of November has not been used in our analysis since the experimental setup had been employed in a modified form and with a different purpose.

where $\tilde{\boldsymbol{s}}(t)$ represents the spatial position within the trajectory of a pedestrian at time t after having applied the Savitzky-Golay filter, and $\Delta t = 1/30$s follows from the cameras aquisition rate.

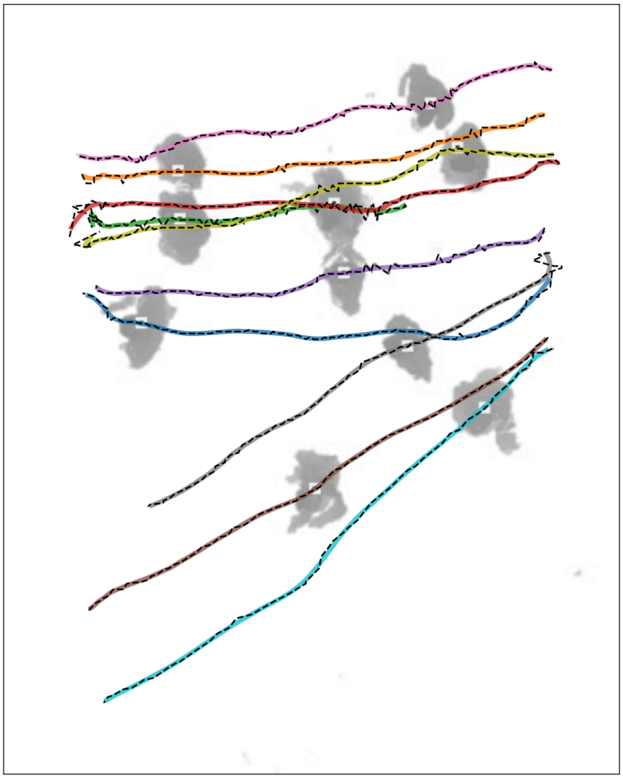

Fig. S2. Snapshot of data recorder by the depth cameras. The black dotted lines represent the trajectories of each pedestrian, obtained by applying the HA-HOG algorithm. Solid lines have been obtained by applying a Savitzky-Golay filter.

Data analysis

Refinement of the dataset In the analysis we have considered only configurations from uni-directional flows. We have dropped all trajectories of pedestrians traveling in the opposite direction with respect to the viewpoint in Fig. 2 in the main text, as well as all trajectories interacting directly or indirectly with them, i.e. both being present at the same time in at least one frame, or sharing a frame with a pedestrian who has previously interacted with a trajectory going in the opposite direction.

Although the bike lane adjacent to the experimental setup was supposedly closed to traffic during the festival hours, cyclists and runners were still present and able to access it from the street. In order to drop from the dataset cyclists, runners, as well as people standing still under the area covered by our sensors, we have retained trajectories with instantaneous velocities $v(t)$ in the interval of $[0.05, 2.9]$ m/s and average velocity $\langle v \rangle$ of $[0.15, 1.5]$ m/s.

Throughout the week, we have collected $192,229$ individual trajectories. Following the above discussion, the analysis retains $101,867$ among these trajectories.

Data de-correlation The calculation of the average number of pedestrians walking in each path as a function of the global pedestrian count requires extra care due to presence of strong correlations between consecutive frames.

For this reason, in our analysis we have taken into consideration only configurations at least 4 seconds apart from each other. This value is larger than the average time duration of a single trajectory, which in our data corresponds to ≈ 3.9 seconds.

In Fig. S3 we show the number of frames contributing to the statistics of pedestrians walking in path A and B as a function of the global pedestrian count. Dots represent the full dataset, whereas triangles represent the uncorrelated dataset.

For the calculation of the local and global velocity fundamental diagram we have made use of the full dataset, since, in this case, time correlations do not introduce biases in the analysis.

Estimation of fluctuations in the velocity fundamental diagram In Eq.3 in the main text we define an additive noise term ϵ for the fundamental velocity diagram, with ϵ drawn from a Gaussian distribution with zero mean and variance $\sigma = 0.15$. We have estimated σ from the the average fluctuations of the local pedestrian velocity in dependence of N_{A} and N_{B}, as reported in Fig. S4.

In Fig. S4, we show the average fluctuations of the local pedestrian velocity in dependence of N_{A} and N_{B}. The plot shows the average standard deviation from the (local) average velocity, computed on frames featuring the same number of pedestrians respectively in path A and path B. We observe that, within error bars, σ is constant and independent of N_{A} and N_{B}.

Comparison with a different routing policy

In the main text we have presented numerical results making use of a variational principle implementing a policy in which pedestrians arrange themselves in order to minimize the overall traversal time. This policy is de facto equivalent to imposing the minimization of the average traversal time.

We here consider an alternative policy, in which pedestrians perform routing choices by minimizing the worst case scenario, i.e. the traveling time of the person that takes the longest to

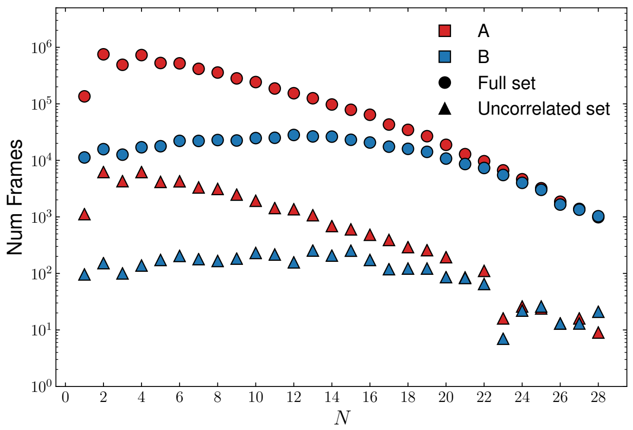

Fig. S3. Number of frames contributing to the statistics of pedestrians walking in path A and B as a function of the global pedestrian count. Dots represent the full dataset, which we use to compute statistics on (and related to) the velocity of pedestrians. Triangles represent the dataset consisting of uncorrelated configurations, taken at least 4 seconds apart from each other, which we use to compute statistics on (and related to) the pedestrian count. The plot gives an indication of the statistical resolution of our analysis.

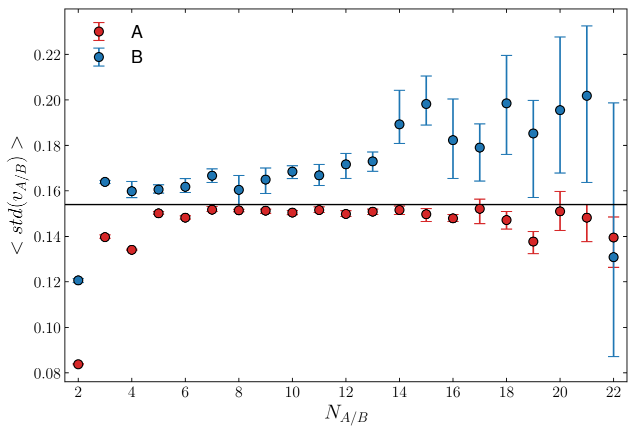

Fig. S4. Average fluctuations of the local pedestrian velocity in dependence of N_{A} and N_{B}, calculated from the experimental data. The plot shows the average standard deviation from the (local) average velocity, computed on frames featuring the same number of pedestrians respectively in path A and path B. The black line represents the global average value of $\sigma = 0.154$.

reach destination. The discomfort functional in this case reads as

$$\mathcal{L} = \max_i \tau^{(i)}_{J_i}. \quad \text{(S2)}$$

Even in this case, when neglecting stochastic terms the model reduces to a Hughes-like form [S3], with the analytic solution $N_{\mathrm{A}} = N_{\mathrm{A}}(N)$ defined by the relation

$$\frac{L_{\mathrm{A}}}{v_{\mathrm{A}}(N_{\mathrm{A}})} = \frac{L_{\mathrm{B}}}{v_{\mathrm{B}}(N_{\mathrm{B}})} = \lambda_g \frac{L_{\mathrm{A}}}{v_{\mathrm{B}}(N_{\mathrm{B}})} \quad , \quad \text{(S3)}$$

from which it directly follows

$$\lambda_g = \frac{v_{\mathrm{B}}(N_{\mathrm{B}})}{v_{\mathrm{A}}(N_{\mathrm{A}})} \quad . \quad \text{(S4)}$$

We remark that λ_g is equal to the velocity ratio between path B and A, at variance with the minimum-overall policy reported

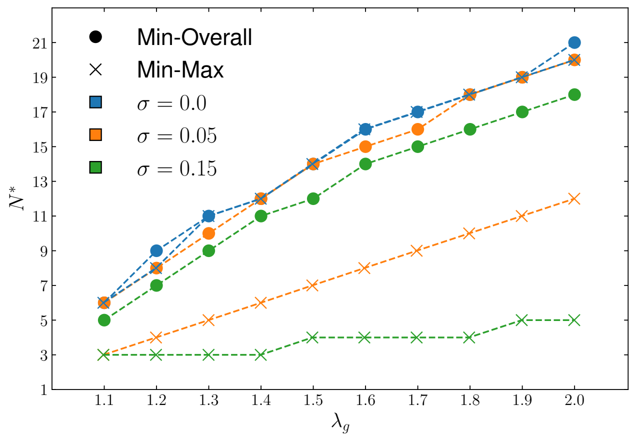

Fig. S5. Comparison of two routing policies, the min-overall policy presented in the main text, and the min-max policy included in the supplementary material. The plot shows the threshold value N^* calculated from simulations using different values of λ_g. We highlighting the effect of introducing fluctuations (σ) on pedestrians walking speed. While the two policies provide very similar results in the deterministic case ($\sigma = 0$), we observe that strong differences arise when including fluctuations. In particular, for values of σ comparable with those observed in the experimental data ($\sigma = 0.15$), the min-max policy favors the use of path B at a much earlier stage with respect to what observed in the experiment. (cf. Fig. 7 in the main text).

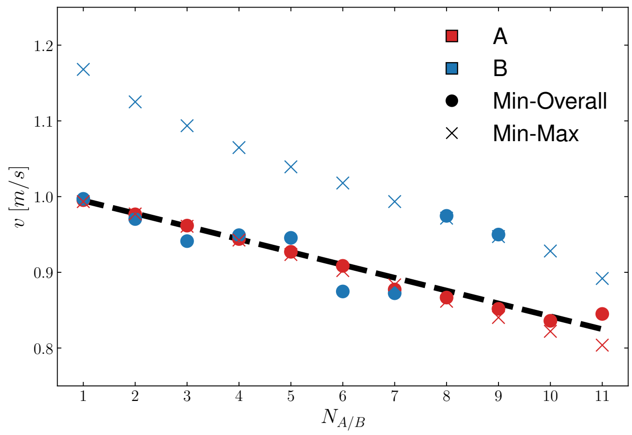

Fig. S6. Local velocity diagram calculated from two simulations with $\sigma = 0.1$, $\lambda_g = 1.7$ for the min-max policy, and $\lambda_g = 1.3$ for the min-overall policy. The black dotted line shows the linear fit of the experimental data, which is used in simulations to determine the velocity of pedestrians. The plot shows that the min-max policy introduces a systematic selection mechanism, which leads to placing fast walkers in path B, a feature which does not emerge from the experimental data.

in the main text where λ_g was instead put in relation with the square of the velocity ratio. We take this aspect into account in the comparison of the two different policies. In Fig. S5 we plot the results of the policy minimizing the worst case scenario (Eq. S2, "min-max" henceforth) in correspondence of the square of value of λ_g used in simulations, in order to make it directly comparable with the min-overall policy.

In Fig. S5 we compute the threshold value N^* for different values of λ_g, comparing the two different policies. When considering the deterministic case (respectively Eq. S3 and Eq.16 in the main text) we observe that the two policies provide

very similar results. However, strong differences arise when including fluctuations in the local velocity diagram. When pedestrians with different walking speed are present, the min-max policy favors the use of path B at a much earlier stage with respect to the min-overall policy; crucially, the latter provides a more accurate description of the experimental data, since with $\lambda_g = 1.33$ and $\sigma = 0.15$ we correctly reproduce the transition at N^* (see again Fig. 7 in the main text). Reproducing these results with the min-max policy, by accounting for the fluctuations observed in the velocity diagram, would require an (artificially) larger value of λ_g.

In Fig. S6 we present a second evidence in support to the fact that the min-overall policy provides a more accurate description of the experimental data. In the figure we show a sort of self-consistency check, by calculating the local velocity diagram from two simulations with $\sigma = 0.1$, $\lambda_g = 1.7$ for the min-max policy, and $\lambda_g = 1.3$ for the min-overall policy. The black dotted line represents the linear fit of the experimental data, used in simulations to determine the velocity of pedestrians. The plot shows that the min-max policy introduces a systematic selection mechanism, which leads to placing fast walkers in path B, a feature which does not emerge from the experimental data (cf. Fig. 4a in the main text).

Relationship between pedestrian count and density

In this work we have considered a time-independent modeling approach, which has allowed us to neglect complex time correlations whose comprehension would have required much more statistics. Within this framework we have found convenient to take into consideration for our analysis the pedestrian count N. As already stated in the main text, this quantity can be put in relationship with the density ρ via

$$\rho = \frac{N}{A}, \tag{S5}$$

with A the measurement area.

The overall area covered by our depth cameras (Fig. 2) consists of approximately $A \approx 28\ \mathrm{m}^2$. However, using such value for A would lead to an underestimation of the density since the measured trajectories do not uniformly distribute on the measurement area because of the geometry and typical flow conditions. Therefore, we compute the density with respect to an effective area A_{ref}, shaped after the effective floor usage.

In order to calculate A_{ref} we have applied a threshold to the probability distribution function of the pedestrian positions (2d-histogram in Fig. 1c). This corresponds to the region bounded by the red line in Fig. S7, which represents a domain contributing the 99% of the occupancy probability. This yields $A_{\mathrm{ref}} = 14.67\ \mathrm{m}^2$.

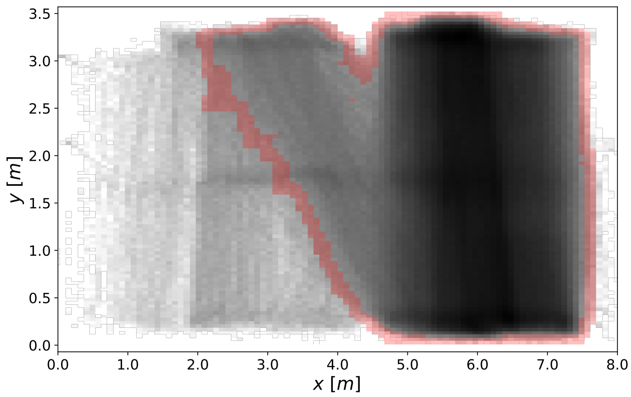

Fig. S7. Boundary (red line) of the reference area (A_{ref}) used in the estimation of the pedestrian density (ρ). The probability distribution function of the pedestrian positions, used in the definition of A_{ref}, is shown in logarithmic scale (grayscale colormap).

References

[S1] Alessandro Corbetta, Werner Kroneman, Maurice Donners, Antal Haans, Philip Ross, Marius Trouwborst, Sander Van de Wijdeven, Martijn Hultermans, Dragan Sekulovski, Fedosja van der Heijden, Sjoerd Mentink, and Federico Toschi. A large-scale real-life crowd steering experiment via arrow-like stimuli. Collective Dynamics, 5:61–68, 2020.

[S2] Alessandro Corbetta, Jasper A. Meeusen, Chung-min Lee, Roberto Benzi, and Federico Toschi. Physics-based modeling and data representation of pairwise interactions among pedestrians. Phys. Rev. E, 98:062310, Dec 2018.

[S3] R. L. Hughes. The flow of human crowds. Annual Review of Fluid Mechanics, 35(1):169–182, 2003.

[S4] Werner Kroneman, Alessandro Corbetta, and Federico Toschi. Accurate pedestrian localization in overhead depth images via height-augmented hog. Collective Dynamics, 5:33–40, 2020.

[S5] Abraham Savitzky and Marcel JE Golay. Smoothing and differentiation of data by simplified least squares procedures. Analytical chemistry, 36(8):1627–1639, 1964.
